# Co-Mn Complex Oxide Nanoparticles as Potential Reactive Oxygen Species Scavenging Agents for Pulmonary Fibrosis Treatment

**DOI:** 10.3390/molecules29215106

**Published:** 2024-10-29

**Authors:** Wuhao Yang, Hui Yuan, Hao Sun, Ting Hu, Yaping Xu, Yan Qiu, Yuhang Li

**Affiliations:** 1 College of Materials, Xiamen University, Xiamen 361005, China; 37420222204264@stu.xmu.edu.cn; 2 State Key Laboratory of Structural Chemistry, Fujian Institute of Research on the Structure of Matter, Chinese Academy of Sciences, Fuzhou 350002, China; xmyuanhui@fjirsm.ac.cn (H.Y.); xmsunhao@fjirsm.ac.cn (H.S.); huting148@163.com (T.H.); 3 Xiamen Key Laboratory of Rare Earth Photoelectric Functional Materials, Xiamen Institute of Rare Earth Materials, Haixi Institutes, Chinese Academy of Sciences, Xiamen 361021, China; 4 Key Laboratory of Functional and Clinical Translational Medicine, Fujian Province University, Xiamen Medical College, Xiamen 361023, China; ypxu@xmmc.edu.cn; 5 School of Medicine, Xiamen University, Xiamen 361102, China; 6 Xiamen Key Laboratory of Chiral Drugs, Xiamen 361102, China

**Keywords:** metal oxide nanoparticles, Co-MnNPs, reactive oxygen species (ROS), pulmonary fibrosis

## Abstract

Idiopathic pulmonary fibrosis (IPF) is a chronic and age-related lung disease that has few treatment options. Reactive oxygen species (ROS) play an important role in the introduction and development of IPF. In the present study, we developed multifunctional Cobalt (Co)–Manganese (Mn) complex oxide nanoparticles (Co-MnNPs), which can scavenge multiple types of ROS. Benefiting from ROS scavenging activities and good biosafety, Co-MnNPs can suppress canonical and non-canonical TGF-β pathways and, thus, inhibit the activation of fibroblasts and the productions of extracellular matrix. Furthermore, the scavenging of ROS by Co-MnNPs reduce the LPS-induced expressions of pro-inflammatory factors in macrophages, by suppressing NF-κB signaling pathway. Therefore, Co-MnNPs can reduce the excessive extracellular matrix deposition and inflammatory responses in lungs and, thus, alleviate pulmonary fibrosis induced by bleomycin (BLM) in mice. Taken together, this work offers an anti-fibrotic agent for treatment of IPF and other ROS-related diseases.

## 1. Introduction

Idiopathic pulmonary fibrosis (IPF) is a chronic and age-related lung disease that affects nearly 5 million persons globally [1]. IPF is characterized by progressive lung scarring, chronic inflammation, and irreversible destruction of the lung architecture [2]. If left untreated, the median survival time of IPF is 2 to 5 years from the time of diagnosis. To date, there is no ideal disease-modifying treatment strategies for IPF [3]. Immunomodulatory therapy using a combination of corticosteroids and azathioprine is the most used treatment for IPF. Unfortunately, a number of adverse consequences, such as poor patient compliance, high toxicity, and limited efficiency, limit their use [4]. Nintedanib and pirfenidone are two anti-IPF agents approved by the US Food and Drug Administration (FDA) in 2014 [5]. However, they only slow down the progression of fibrosis and cannot completely restore or even stabilize lung function [6]. Additionally, their applications were hampered by numerous complications, including diarrhea, increased risk of arterial thrombosis, and gastrointestinal injury [5,7]. Therefore, developing new therapeutic approaches capable of slowing down IPF progression with significant side effects is desirable.

Although the exact cause for IPF remains unknown, oxidative stress, uncontrolled inflammation, and abnormal deposition of extracellular matrix (ECM) have been considered closely related to the progression of IPF [8]. Sustained production of reactive oxygen species (ROS) induced oxidative stress damage to alveolar epithelium, resulting in the activation of pulmonary fibroblasts and immune cells [9,10]. These cells migrate to fibrotic foci, where they release pro-fibrotic mediators, such as transforming growth factor β (TGF-β) and pro-inflammatory factors, e.g., tumor necrosis factor α (TNF-α) [1]. TGF-β acts on fibroblasts, stimulating them to differentiate into myofibroblasts and produce excessive ECM, ultimately resulting in irreversible organ damage and loss of lung function [11]. TNF-α stimulates the activities of key immune cells, such as macrophages, which release additional chemokines and pro-inflammatory cytokines, leading to the expansion of local inflammation and the proliferation of fibroblasts [12]. Moreover, TGF-β and TNF-α also cause an excess of ROS. These innate oxidative mediators further damage alveolar epithelial cells and exacerbate pulmonary inflammatory and fibrotic responses [1,13].

Therefore, antioxidant therapy targeting the key pathogenesis of IPF represents an effective approach for attenuating pulmonary fibrosis [13]. In the past decade, many antioxidant small molecules have been developed, and some of them, such as N-acetylcysteine, have even been used in the clinical treatment of IPF [14]. However, the low therapeutic effects and poor pharmacokinetics restrict their application. Recently, antioxidant therapies using ROS-scavenging nanomaterials have been reported to overcome this limitation [13]. Nanomaterials, including Au, Se, Vanadium carbide and fullerene nanoparticles, with antioxidant enzyme-like catalytic characteristics capable of scavenging ROS, have been demonstrated as promising therapeutic strategies for IPF [15,16,17,18,19]. However, insufficient ROS scavenging catalytic activity, intrinsic toxicity, and complicated preparation process extremely limit the therapeutic effect of many reported nano enzymes [13]. Manganese (Mn)-based nanomaterials have received multitudinous concern as prospective agents for scavenging pathological ROS and treatment of ROS-related diseases [20,21,22], including rheumatoid arthritis [23], Alzheimer’s disease (AD) [24], diabetic wound healing [25], and inflammatory bowel disease [26]. So far, Mn-based nanomaterials have not been explored as an anti-pulmonary fibrosis therapeutic approach. Treatment of pulmonary fibrosis with Mn-based nanomaterials has the potential to achieve profound therapeutic efficacy.

**Figure 1:**
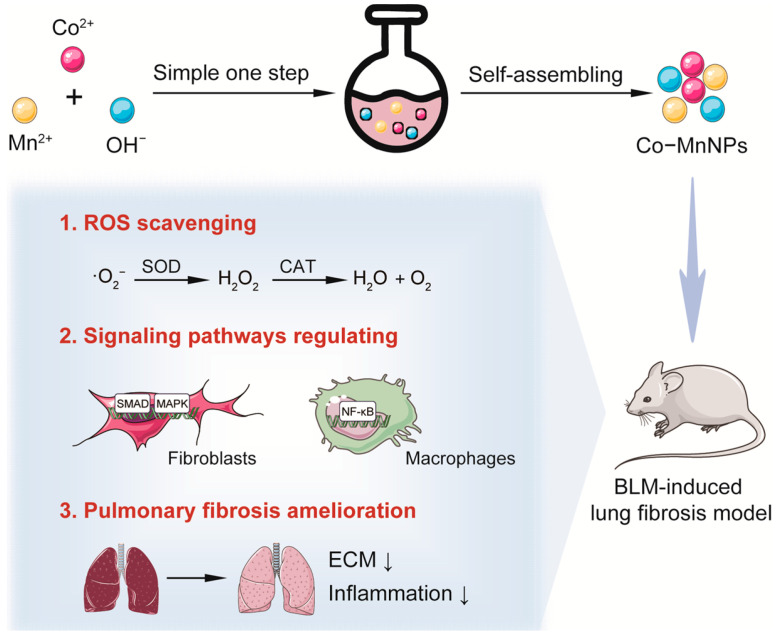
Schematic diagram of the preparation of Co-MnNPs with ROS scavenging activity for treating lung fibrosis in mice.

It has been reported that the introduction of Cobalt (Co) into Mn-based nanomaterials can produce oxygen vacancies, enhancing the internal electron transfer efficiency of materials [27]. Moreover, many Cobalt (Co)-based nanomaterials have demonstrated potent ROS scavenging activities [28,29,30,31,32]. It is possible that the introduction of Co into Mn-based nanomaterial may enhance its ROS scavenging activity. Furthermore, scavenging ROS with a Co-Mn-based nanomaterial may block the activation of key fibrosis signaling in the development of IPF and prevent inflammation and extracellular matrix abnormality, thereby alleviating the progression of pulmonary fibrosis. Based on this hypothesis, in the present study, we developed a multifunctional Co-Mn complex oxide nanoparticle (Co-MnNPs), with potent and broad-spectrum ROS scavenging activity. Due to the ROS scavenging properties and good biocompatibility, Co-MnNPs can suppress TGF-β-induced human fetal lung fibroblast (HFL) activation, inhibiting the production of extracellular matrix. Additionally, Co-MnNPs can reduce LPS-induced inflammatory reactions in RAW264.7 macrophages with decreased expressions of pro-inflammatory factors. Therefore, Co-MnNPs were able to attenuate pulmonary fibrosis induced by bleomycin (BLM), reducing the excessive extracellular matrix deposition and pro-inflammatory factor expression. Mechanistically, Co-MnNPs suppressed fibrotic and inflammatory responses through NF-κB and TGF-β pathways (Figure 1). In conclusion, the present study suggests that Co-MnNPs are an effective anti-fibrotic agent for pulmonary fibrosis.

## 2. Results and Discussions

### 2.1. Synthesis and Characterization of Co-MnNPs

**Figure 2:**
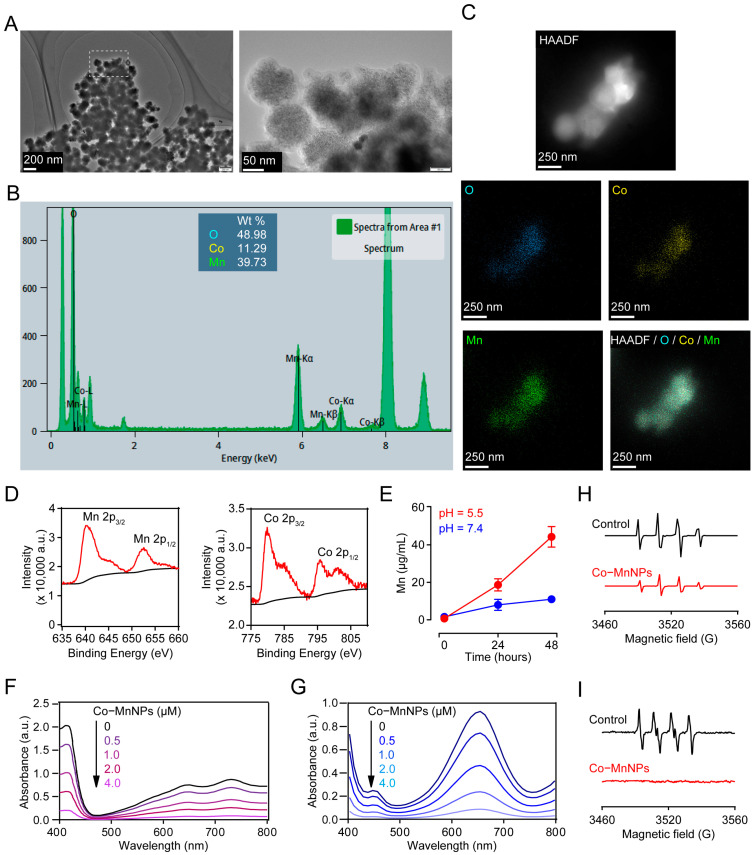
The synthesis and characterizations of Co-MnNPs. (**A**) TEM and (**B**) EDS of Co-MnNPs. (**C**) Elemental mapping images of Co-MnNPs. (**D**) XPS analysis of Co-MnNPs. (**E**) Co-MnNPs was incubated in PBS solutions (pH = 5.5 and 7.4) for 2 days, and following centrifugation, the supernatant was analyzed by ICP-MS. (**F**) UV−vis spectra of ABTS• and (**G**) ox-TMB solution in different concentrations of Co-MnNPs. ESR spectra of the solution of (**H**) DMPO/•OH and (**I**) BMPO/O_2_•^−^ in the presence or absence of Co-MnNPs.

Co-MnNPs were synthesized by simply mixing NaOH with Mn (OAc)_2_ and Co(NO_3_)_2_ in the presence of L-ascorbic acid (AA) as a reductant and stabilizing reagent in a one-pot reaction (Figure 1). The synthesis method is simple and feasible for large-scale preparation. We then characterized the structure of Co-MnNPs by a transmission electron microscope (TEM). The obtained Co-MnNPs exhibited spherical morphology with an average size of 100 nm (Figure 2A). In addition, as shown in Figure 2B,C, the elemental mapping images of Co-MnNPs showed a uniform distribution of Mn, Co, and O. Energy dispersive X-ray spectroscopy (EDS) analysis further demonstrated that the percentage of Co and Mn in Co-MnNPs was 14.82% and 12.98%, respectively (Figure 2B). The composition of Co-MnNPs was also studied by XPS. As shown in Figure 2D, the peaks at 780 eV and 797 eV were assigned to Co, while at 640 eV and 652 eV, the typical peaks were assigned to Mn. These results demonstrated the formation of Co-MnNPs. Additionally, to exclude the possibility that the synthesized Co-MnNPs may be capped with AA, we synthesized Co-Mn particles (Co-Mn) without AA and examined the obtained Co-Mn, Co-MnNPs, and AA with thermogravimetric analysis (TGA). As shown in Figure S2, AA began to decompose at 191 °C, and the weight loss percentage was nearly 80% at 800 °C. However, this was not more than 5% weight loss of Co-MnNPs and Co-Mn at 800 °C. These results indicated that the obtained Co-MnNPs contained no more than 5% AA.

Inhalation of nondegradable particles, such as crystalline silica, may cause persistent damage to the lung tissues, leading to the incurable pulmonary fibrosis [33]. Thus, we tested whether Co-MnNPs can be degraded in biological conditions. Co-MnNPs were incubated in PBS solutions with different pH at 37 °C for 48 h. The concertation of dissolved Mn was determined by inductively coupled plasma mass spectrometry (ICP-MS). The dissolution rate of Co-MnNPs in the pH = 7.4 solutions was slow, and no notable changes in the dissolved Mn levels were observed during 48 h incubation (Figure 2E). However, in the pH = 5.5 solutions, the concertation of dissolved Mn was greatly increased after incubation for 48 h (Figure 2E). These data indicate that Co-MnNPs may be degraded in lysosomes after intake by cells, where the pH is 4–5.5. However, the PBS buffers are not equal to plasma and pulmonary tissue fluid, which are protein-rich solutions. We could not exclude the possibility that the degradation of nanoparticles is affected by the protein in these solutions through interaction with nanoparticles and the solvent. Future studies should be carried out to confirm this action [34]. In addition, to study the reason for the difference on the dissolution rate of Co-MnNPs at pH 5.5 and 7.4, we assessed the colloidal stability of nanoparticles at pH 5.5 and 7.4 by particle dispersion index (PDI) assay. As shown in Table S1, the PDI values of Co-MnNPs solution at pH 5.5 were not significantly changed over 48 h, indicating that the colloidal solution of Co-MnNPs at pH 5.5 is stable. The PDI values of Co-MnNPs solution at pH 7.4 were not changed within 24 h, but greatly increased after 36 h, indicating that Co-MnNPs were aggregated at pH 7.4 after 36 h. It is possible that the aggregation at pH 7.4 decreased the specific surface area of Co-MnNPs and slowed down the dissolution rate.

We next evaluated the ROS scavenging capability of Co-MnNPs by using azino-bis(3-ethylbenzothiazoline-6-sulfonic acid) (ABTS) and 3′3′5′5′-tetramethylbenzidine hydrochloride dehydrate (TMB) assays. ABTS can interact with potassium persulfite and generate reactive radical cation ABTS• with a peak absorbance of 734 nm [35]. As shown in Figure 2F, the generation of ABTS• was dose-dependently scavenged by Co-MnNPs. Meanwhile, to exclude the possibility that Co-MnNPs scavenge ROS by simple adsorption of ABTS radicals on nanoparticles, we used Trolox as the positive control. As shown in Figure S3, Trolox (1 μM) effectively reduced the production of ABTS radicals, indicating that the decrease in the ABTS radicals signal based on the antioxidant properties of materials. Similarly, TMB can be oxidized by •OH, generated by the Fenton reaction of FeSO_4_ and H_2_O_2_, resulting in the oxidized type of TMB (ox-TMB) with a characteristic absorbance at 650 nm [36]. Treatment with Co-MnNPs dose-dependently decreased ox-TMB (Figure 2G). These data demonstrate the ROS scavenging capability of Co-MnNPs. Moreover, to exclude the effect of residual AA, we also tested bare Co-Mn and AA (5% of Co-MnNPs weight) in the ABTS and TMB assays. As shown in Figure S4, bare Co-Mn particles exhibited similar anti-oxidative effects in ABTS and TMB assays, while AA showed no effects. These results indicated that the ROS scavenging capability of Co-MnNPs is not derived from residual AA.

Furthermore, we investigated the species of ROS scavenged by Co-MnNPs using electron spin resonance (ESR) spectra. The •OH was generated by a typical Fenton reaction with FeCl_2_ and H_2_O_2_ and captured with 5,5-dimethyl-1-pyrroliline nitrogen oxide (DMPO) to form DMPO/•OH [19]. The O_2_•^−^ was produced by a classic enzymatic reaction with xanthine oxidase and hypoxanthine and captured with 5-tertbutyl-5-methyl-1-pyrroliline nitrogen oxide (BMPO) to form BMPO/O_2_•^−^ [19]. As indicated in Figure 2H,I, ESR spectra showed that treatment with Co-MnNPs reduced the signals of both DMPO/•OH and BMPO/O_2_•^−^, demonstrating the activity of Co-MnNPs to scavenge •OH and O_2_•^−^. When combined, these data confirm the ROS scavenging capability of Co-MnNPs, which could reduce •OH and O_2_•^−^ and can be used as antioxidant agents for the treatment of pulmonary fibrosis.

### 2.2. Co-MnNPs Restrained TGF-β-Induced Activation of NIH/3T3

**Figure 3:**
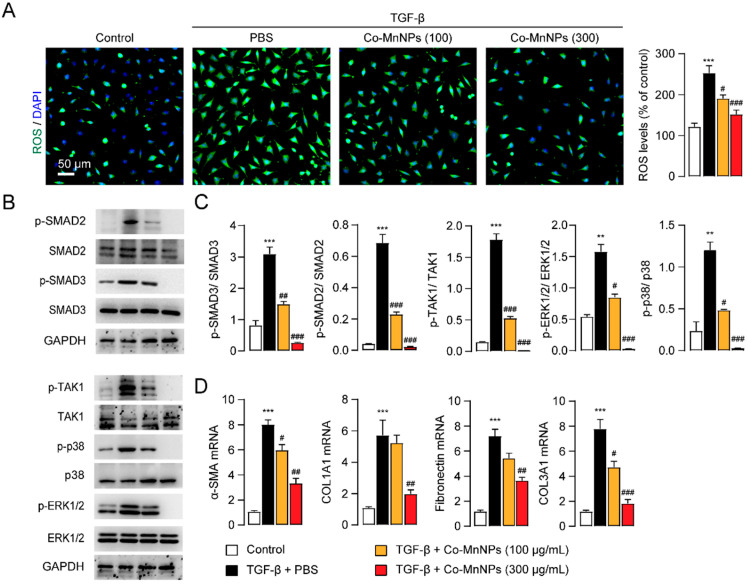
Co-MnNPs inhibited TGF-β1-induced differentiation of HFLs through scavenging ROS. HFLs cells were exposed to PBS control or TGF-β (5 ng/mL) for 30 min, followed by treatment with Co-MnNPs (100 μg/mL), Co-MnNPs (300 μg/mL), or their PBS for 24 h. (**A**) Representative confocal images and quantification of ROS in HFLs cells. (**B**,**C**) Expressions of p-SMAD2, p-SMAD3, SMAD2, SMAD3, p-TAK1, TAK1, p-p38, p38, p-ERK1/2, and ERK1/2 in NIH/3T3 were presented by Western blotting. (**D**) mRNA levels of COL1A1, COL3A1, α-SMA, and fibronectin in HFL cells presented by PCR. **, *p* < 0.01, ***, *p* < 0.001 relative to control; #, *p* < 0.05, ##, *p* < 0.01, ###, *p* < 0.001 relative to PBS group, n = 3 per group.

Fibroblasts are key effector cells in the development of IPF [1]. The cell viability of NIH-3T3 and RAW264.7 cells after 24 h incubation with Co-MnNPs (300 µg/mL) was higher than 90%, indicating that Co-MnNPs have lower cytotoxicity to normal cells (Figure S6). We then investigated the influence of Co-MnNPs on oxidative stress in NIH/3T3 fibroblasts. TGF-β stimulation induced the overproduction of ROS in NIH/3T3 cells, while Co-MnNPs dose-dependently reduced the generation of ROS (Figure 3A). The activation of fibroblasts is primarily regulated by canonical TGF-β signaling pathways, such as SMAD2 and SMAD3, and non-canonical pathways involving TGF-β-activated kinase 1 (TAK1), ERK1/2, and p38 [8,37]. We then studied the influence of Co-MnNPs on the canonical TGF-β signaling. Western-blotting studies suggested that stimulation of NIH/3T3 fibroblasts with TGF-β significantly increased the phosphorylation of SMAD2 and SMAD3 (Figure 3B,C). However, treatment with Co-MnNPs dose-dependently decreased such increases (Figure 3B,C). TGF-β stimulation also induced the phosphorylation of TAK1 and subsequent activation of ERK1/2 and p38 signaling, the key players in non-canonical TGF-β signaling [37,38], whereas the administration of Co-MnNPs suppressed the activation of these signaling molecules in TGF-β-treated NIH/3T3 cells (Figure 3B,C). Consistent with the results from Western blotting studies, immunofluorescent studies showed that levels of phosphorylated TAK1 (p-TAK1) and α-SMA, a biomarker of activated fibroblasts [9], were greatly increased in NIH/3T3 cells following treatment with TGF-β (Figure S7). These increasements were dose-dependently reduced by Co-MnNPs (Figure S7). Additionally, we evaluated the influence of Co-MnNPs on the functions of fibroblasts. TGF-β stimulation resulted in the upregulation of α-SMA, and the ECM components, including COL1A1, COL3A1, and fibronectin in NIH/3T3 cells [1], suggesting that NIH/3T3 fibroblasts underwent activation and differentiation, whereas Co-MnNPs hindered these cellular responses (Figure 3D). Moreover, to exclude the possibility that Co-MnNPs reduce the activation of fibroblasts by adsorption of TGFβ, we cultured NIH/3T3 fibroblasts with MSN (300 μg/mL), a well-known nanomaterial with broad inner surfaces and volumes, for 24 h at 37 °C. As shown in Figure S5, treatment with MSN could not affect ROS generation in NIH/3T3. Additionally, MSN had no effects on the mRNA expressions of pro-fibrotic factors. These results indicated that Co-MnNPs reduce the activation of fibroblasts through scavenging ROS but not adopting TGFβ. Furthermore, treatment with bare Co-Mn particles but not AA (5% of Co-MnNPs weight) reduced ROS production and the expression of pro-fibrotic mediators in NIH/3T3 (Figure S5), indicating the anti-fibrotic action of Co-MnNPs is not derived from residual AA. Collectively, Co-MnNPs restrained activation of fibroblasts through scavenging ROS and attenuating the canonical and non-canonical TGF-β signaling.

### 2.3. Co-MnNPs Reduced LPS-Induced Inflammation in RAW264.7 Macrophages

**Figure 4:**
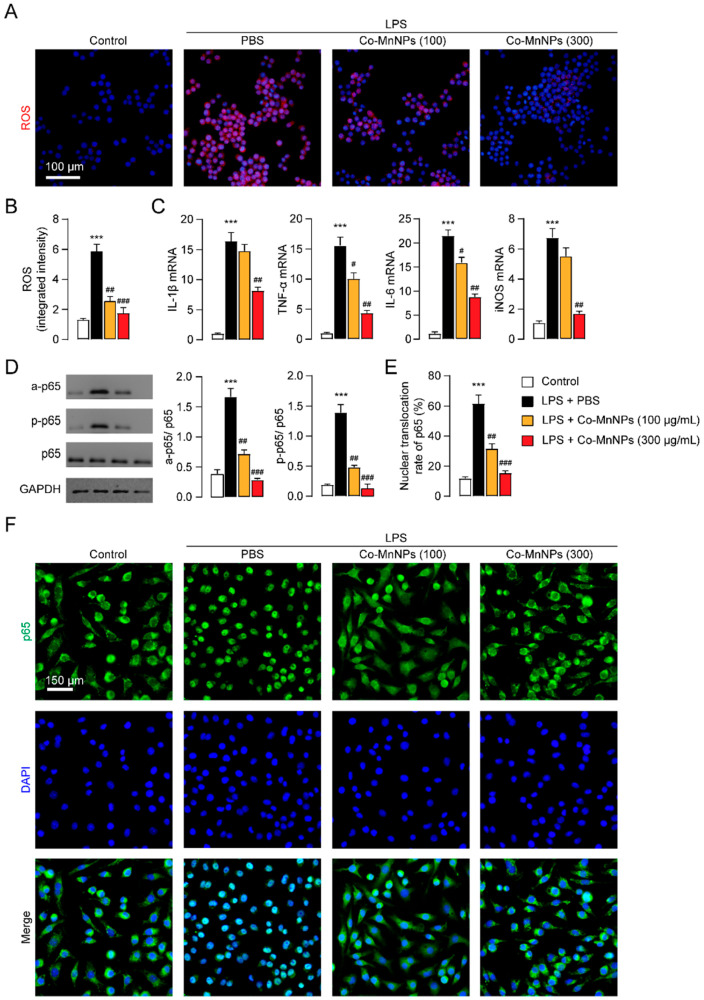
Co-MnNPs inhibited LPS-induced inflammation in RAW264.7 macrophages through scavenging ROS. RAW264.7 cells were exposed to PBS control or LPS (100 ng/mL) for 30 min, followed by treatment with Co-MnNPs (100 μg/mL), Co-MnNPs (300 μg/mL), or their PBS for 24 h. (**A**) Representative confocal images and (**B**) quantification of ROS in RAW264.7 cells. (**C**) mRNA levels of IL-1β, TNF-α, IL-6, and iNOS presented by PCR. (**D**) Expressions of a-p65, p-p65, and p65 presented by Western blotting. (**E**) Quantification of p65 nuclear translocation in (**F**). (**F**) Immunofluorescent staining of p65 in RAW264.7 cells. ***, *p* < 0.001 relative to control; #, *p* < 0.05, ##, *p* < 0.01, ###, *p* < 0.001 relative to PBS group, n = 3 per group.

Macrophages are another important effector cell in the development of IPF [10]. We then studied the influence of Co-MnNPs on LPS-induced inflammation in RAW264.7 macrophages. Stimulation with LPS induced the overproduction of ROS in RAW264.7 cells, while Co-MnNPs could dose-dependently reduce the generation of ROS (Figure 4A,B). Furthermore, Co-MnNPs also decreased LPS-induced persistent productions of pro-inflammatory mediators, like IL-1β, TNF-α, IL-6, and iNOS (Figure 4C), indicating the potent anti-inflammatory effects of Co-MnNPs. Meanwhile, MSN had no such effects (Figure S5). Furthermore, treatment with bare Co-Mn particles but not AA (5% of Co-MnNPs weight) reduced ROS production and the expression of pro-inflammatory factors in RAW264.7 cells, respectively (Figure S5). These data indicate the anti-inflammatory action of Co-MnNPs is not derived from residual AA. NF-κB signaling is the key pathway controlling the inflammatory reaction in macrophages [10,39]. We next examined whether scavenging ROS by Co-MnNPs can regulate the activation of NF-κB signaling. As shown in Figure 4D, treatment of RAW264.7 macrophages with LPS promoted the phosphorylation and acetylation of p65, a critical process in the activation of the NF-κB pathway [40]. Following the phosphorylation and acetylation, translocation of p65 from the cytoplasm to the nucleus is also observed (Figure 4E,F). However, treatment with Co-MnNPs significantly inhibited the LPS-induced p65 activation and translocation from the cytoplasm to the nucleus (Figure 4D,F). These findings indicate that Co-MnNPs can potentially limit the inflammation induced by LPS in macrophages by scavenging ROS and suppressing the NF-κB pathway.

### 2.4. Co-MnNPs Alleviated Pulmonary Fibrosis in Mice

**Figure 5:**
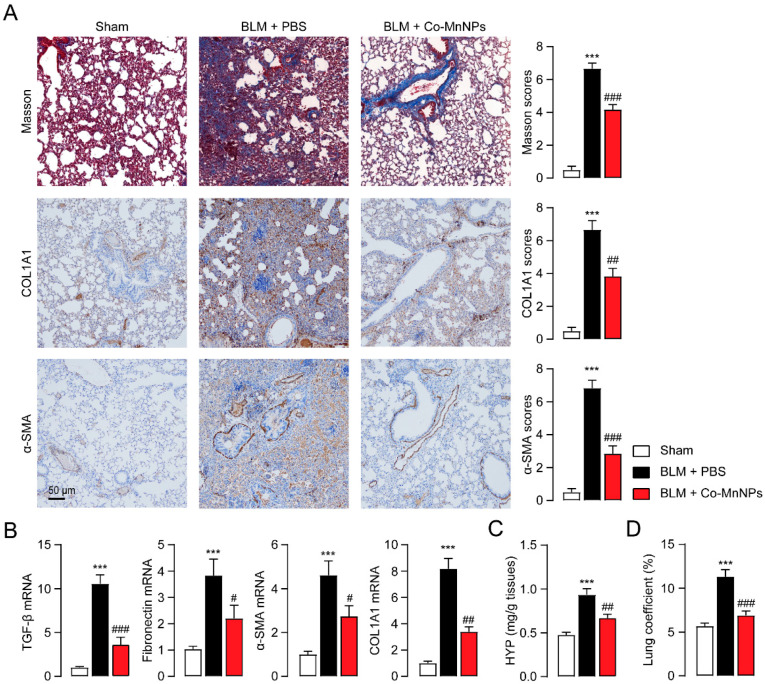
Co-MnNPs prevented lung fibrosis in mouse. (**A**) Masson’s trichrome staining and immunochemical staining for COL1A1 and α-SMA in lung sections. (**B**) mRNA levels of TGF-β, COL1A1, α-SMA, and fibronectin in lungs were presented by PCR. (**C**) The content of hydroxyproline (HYP) and (**D**) the lung coefficient in lung tissues. ***, *p* < 0.001 relative to sham group; #, *p* < 0.05, ##, *p* < 0.01, ###, *p* < 0.001 relative to BLM + PBS group, n = 5 per group.

Based on the promising in vitro findings of Co-MnNPs, we further determined whether Co-MnNPs can attenuate BLM-induced lung fibrosis in mice. The mice were administered BLM (3 mg/kg) and subsequently intratracheally injected with Co-MnNPs once every week for 4 weeks. Excessive ECM deposition is an important feature during IPF [1]. Examination of Masson’s trichrome staining revealed that Co-MnNPs alleviated the dense collagen accumulation caused by BLM, specifically in the vicinity of the bronchial wall and alveolar septum area (Figure 5A). Immunochemistry staining showed that Co-MnNPs similarly reduced protein expression of α-SMA and COL1A (Figure 5A). Consistently, PCR results indicated that Co-MnNPs also decreased mRNA expression of TGF-β, α-SMA, fibronectin, and COL1A in the lungs of a BLM-induced mice model (Figure 5B). Furthermore, administration of Co-MnNPs resulted in a reduction in the expression of hydroxyproline (HYP), an amino acid that is specific to collagen (Figure 5C). Additionally, the increased lung coefficient caused by BLM in mice was reversed by administration of Co-MnNPs (Figure 5D). These findings indicate that Co-MnNPs can potentially limit the fibrosis responses induced by BLM in mice.

### 2.5. Co-MnNPs Reduced Inflammation in Mice with Pulmonary Fibrosis

**Figure 6:**
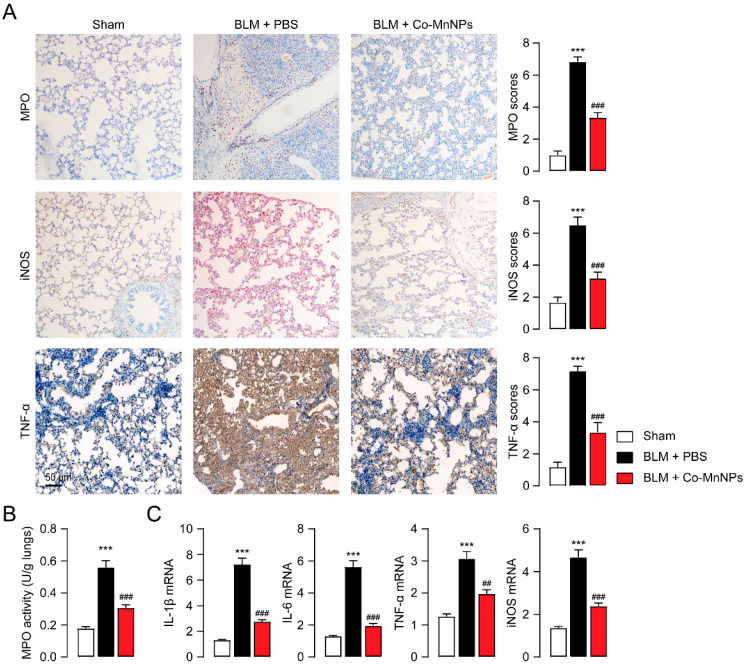
Co-MnNPs prevented pulmonary inflammation in mouse. (**A**) Immunochemical staining for iNOS, MPO, and TNF-α in lungs. (**B**) MPO activity in lung tissues. (**C**) mRNA levels of IL-1β, TNF-α, IL-6, and iNOS presented by PCR. ***, *p* < 0.001 relative to sham group; ##, *p* < 0.01, ###, *p* < 0.001 relative to BLM + PBS group, n = 5 per group.

Furthermore, we investigated whether Co-MnNPs could mitigate pulmonary inflammation in addition to enhancing ECM deposition. The results of the immunohistochemistry assay revealed that BLM caused a noticeable rise in the expression level of MPO, a biomarker for activated neutrophils [41], as well as iNOS and TNF-α (Figure 6A), crucial mediators linked to inflammation and fibrosis [42,43]. Nonetheless, Co-MnNP administration resulted in a marked decrease in the expression of these proteins in the lungs (Figure 6A). Moreover, the MPO activity of the fibrosis tissue was decreased by Co-MnNPs (Figure 6B). Consistently, PCR results indicated that Co-MnNPs suppressed the production of MPO, iNOS, and TNF-α induced by BLM (Figure 6C). In addition, treatment with Co-MnNPs did not induce detectable histological changes in the major organs (including heart, liver, spleen, lungs, and kidneys) of mice (Figure S1), indicating the high biosafety of Co-MnNPs. These data indicate that scavenging ROS with Co-MnNPs reduces inflammation in mice with pulmonary fibrosis.

### 2.6. Co-MnNPs Suppressed TGF-β Pathways in the BLM-Induced Pulmonary Fibrosis Mouse Model

**Figure 7:**
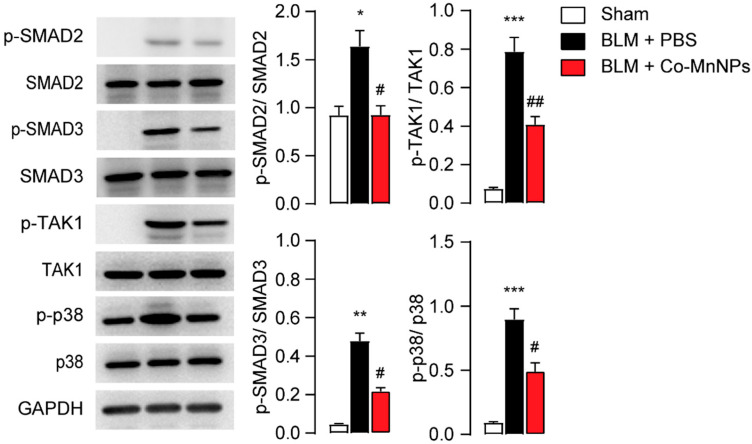
Co-MnNPs suppressed canonical and non-canonical TGF-β signaling in mice. Expression of p-SMAD2, p-SMAD3, SMAD2, SMAD3, p-TAK1, TAK1, p-p38, p38, p-ERK1/2, and ERK1/2 in lungs on day 28 were presented by Western blotting. *, *p* < 0.05, **, *p* < 0.01, ***, *p* < 0.001 relative to sham group; #, *p* < 0.05, ##, *p* < 0.01 relative to BLM + PBS group, n = 5 per group.

Finally, we conducted additional analysis to demonstrate whether Co-MnNPs could suppress the progression of fibrotic responses in mice by regulating both canonical and non-canonical TGF-β signaling pathways. Consistent with the outcomes observed in in vitro experiments, compared to the sham control group, the phosphorylation levels of SMAD2, SMAD3, p38 and TAK1 were found to be higher in the lung of mice with pulmonary fibrosis (Figure 7). Co-MnNPs effectively suppressed the expression of p-SMAD2, p-SMAD3, p-TAK1, and p-p38 in the lungs (Figure 7). The obtained data suggest that the use of Co-MnNPs potentially prevents pulmonary fibrosis by regulating TGF-β signaling pathways.

## 3. Materials and Methods

### 3.1. Materials and Instrumentation

Unless specifically mentioned, reagents utilized in this study were sourced from Yuanye Bio-Technology (Shanghai, China). Bleomycin was purchased from Tsbiochem (Cat. #T6116). TEM and EDS images were captured on a field emission high-resolution transmission electron microscope (JEOL, Peabody, MA, USA). UV spectra were recorded with an Agilent Cary 5000 UV-Vis-NIR spectrophotometer (Agilent, Santa Clara, CA, USA). The diameters of Co-MnNPs were measured using the ZetaSizer Nano ZS90 (Malvern, Makvern, UK). The surface analysis of Co-MnNPs was performed using an XSAM800 X-ray photoelectron spectrometer (Kratos, Manchester, UK). The ESR spectra were recorded with an EPR-ELEXSY ELEXSYS-II E500 CW-EPR (Bruker, Ettlingen, Germany).

### 3.2. Preparation and Characterization of Co-MnNPs

A solution of NaOH (1 M, 3 mL) was added to a mixture of Co(NO_3_)_2_ (0.5 mmol), Mn(OAc)_2_ (2.5 mmol), and L-ascorbic acid (2.0 mmol) in distilled water (30 mL). After stirring at 80 °C for 12 h, the resulting solution was centrifuged at 10,000× *g* for 10 min. The pellet was then washed with distilled water (3 × 15 mL) and ethanol (3 × 15 mL) and dried at 50 °C overnight. The morphology of Co-MnNPs was characterized by TEM. The composition of Co-MnNPs was then verified by EDS and XPS.

### 3.3. Stability Study of Co-MnNPs

Co-MnNPs (1 mg) were incubated in 5 mL PBS (pH = 5.5) or 5 mL PBS (pH = 7.4) at 37 °C for 48 h, centrifuged at 10,000× *g* for 10 min at indicated time points, and the resulting supernatants were then analyzed by ICP-MS.

### 3.4. ROS Scavenging Activity of Co-MnNPs

#### 3.4.1. ABTS Assay

The ABTS• radical was prepared by oxidization of ABTS with potassium persulfate. The ABTS• solution was generated by mixing ABTS (37.4 mg) in 30 mL sodium acetate buffer (20 mM, pH = 4.5) with K_2_S_2_O_8_ (7 mg) and stirred at 4 °C in the dark for 10 h. Then, the ABTS• solution (3 mL) was incubated with various doses of Co-MnNPs at 37 °C for 2 min, and the UV-Vis spectra were determined to evaluate the antioxidant properties of Co-MnNPs. The reduction of the ABTS• radical was determined by monitoring its absorption at 734 nm. Mesoporous silica nanospheres (MSN) (300 μg/mL) were used as a control to exclude the possibility that Co-MnNPs reduced the radical generation through adsorption of ABTS or K_2_S_2_O_8_ (Figure S4).

#### 3.4.2. TMB Assay

The •OH was generated by a classical Fenton reaction between Fe^2+^ and H_2_O_2_. The •OH was generated by mixing FeSO_4_ (2.52 mg) in 10 mL deionized water (pH = 7) with 10% H_2_O_2_ (20 mg) and stirred at 37 °C for 5 min. Then, the •OH solution (10 mL) was incubated with various doses of Co-MnNPs at 37 °C for 5 min, followed by reaction with TMB (4.8 mg) in 1 mL deionized water for 2 min. The UV-Vis spectra were determined to evaluate the •OH scavenging properties of Co-MnNPs. The reduction in the oxidized type of TMB (ox-TMB) was determined by monitoring its absorption at 650 nm. Mesoporous silica nanospheres (MSN) (300 μg/mL) were used as the negative control (Figure S4).

#### 3.4.3. ESR Assay

The DMPO/•OH solution was generated by mixing DMPO (0.25 µmol), H_2_O_2_ (0.1 µmol), and FeCl_2_ (0.1 µmol) in 50 µL deionized water for 5 min. To test the scavenging activity of Co-MnNPs on •OH, the DMPO/•OH solution (50 μL) was incubated with Co-MnNPs (100 μg) for 2 min, and the ESR spectra were recorded.

The BMPO/O_2_•^−^ solution was generated by mixing BMPO (1.25 µmol), hypoxanthine (0.05 µmol), diethylenetriaminepentaacetic acid (2.5 nmol), and xanthine oxidase (2 mU) in 50 µL deionized water. To test the scavenging activity of Co-MnNPs on O_2_•^−^, the BMPO/O_2_•^−^ solution (50 μL) was incubated with Co-MnNPs (100 μg) for 2 min, and the ESR spectra were recorded.

### 3.5. Mouse

Vital River (Beijing, China) provided C57BL/6 mice. The mice used in this study were housed in a specific pathogen-free (SPF) environment in the central Laboratory of Xiamen University. All animal studies were carried out in strict compliance with the relevant guidelines and regulations set forth by ARRIVE and approved by the Animal Care and Use Committee of Xiamen University (Approval No. XMULAC20230132). Anesthesia was administered to male C57BL/6 mice (20–25 g) using isoflurane, followed by the intratracheal injection of BLM (0.6 mg in 0.1 mL saline). This procedure was conducted to induce lung fibrosis in mice [44]. The control groups were administered a nasal instillation of a saline solution in an equal volume. Co-MnNPs were suspended with PBS. Co-MnNPs (200 μg) or its PBS solution (0.1 mL) were intraperitoneally injected (i.p.) once every week for 4 weeks. At 28 days after BLM or saline instillation, mice were sacrificed and lung tissues were collected. The lung coefficient could be determined by the following equation: wet lung weight (in grams)/body weight of the mouse (in kilograms) [44]. The right lung was frozen until further use for RNA preparation and protein isolation. An MPO assay kit (Solarbio, Beijin, China, BC5710) was employed to determine the activity of MPO, and the manufacturer’s instructions were followed during the testing process [44].

### 3.6. Histology

After being fixed in a solution containing 4% paraformaldehyde, the left lung was embedded in paraffin and sectioned. The sections were then stained to measure the amounts of inflammation and collagen buildup using Masson’s trichrome staining and H&E staining. Fibrotic changes in the area were evaluated based on the Ashcroft scale. Masson’s scores were determined by examining 3 randomly selected and non-overlapping fields in each section of the lung. Two independent blind investigators scored each specimen [37,44].

### 3.7. Immunofluorescent Staining

To demonstrate the result of the Western blot assay, we further investigate the non-canonical TGF-β signaling in situ by cell immunofluorescence staining. Cells were seeded and cultured on sterilized glass coverslips. NIH/3T3 fibroblasts and RAW264.7 cells were subjected to three washes with PBS, followed by fixation on coverslips with 4% paraformaldehyde for 20 min and rinsed twice with PBS. Subsequently, the cells were treated with 1% Triton X-100 for 10 min. After two hours of blocking with 5% BSA in 100 mL, the cells were treated with primary antibodies at 4 °C for the entire night. The primary antibodies were anti-phospho-TAK1 (CST, Shanghai, China, Cat. #109404), anti-α-SMA (Proteintech, Wuhan, China, Cat. #55135-1-AP), and anti-p65 (CST, Cat. #6956). The cells were exposed to secondary antibodies tagged with fluorescence for 120 min at 25 °C. The secondary antibodies used were goat anti-rabbit (Proteintech, Cat. #SA00013) and goat anti-rat (Proteintech, Cat. #10285-1-AP). Finally, glass coverslips were placed onto slides and mounted using a mounting medium containing 4′,6-diamidino-2 phenylindole (DAPI) (Vector Lab). The images were captured with confocal microscopy (Olympus, Tokyo, Japan) [45].

### 3.8. Immunohistochemistry (IHC) Staining

To further demonstrate the therapeutic effects of Mn-CoNPs on IPF, we also study the protein levels of pro-fibrotic factors by IHC staining in animals. Paraffin sections underwent a series of procedures including baking, dewaxing, rehydration in graded alcohol, and antigen retrieval by heating sections in sodium citrate buffer (10 mM, pH 6). To prevent non-specific binding, the sections were exposed to 3% H_2_O_2_ for 5 min, washed with 3,3′-Diaminobenzidine (DAB) rinse buffer, and treated with 3% BSA for 60 min. The primary antibodies: anti-α-SMA (Proteintech, Cat. #55135-1-AP), anti-COL1A1 (Proteintech, Cat. #67288-1-Ig), anti-MPO (Proteintech, Cat. #22225-1-AP), anti-iNOS (CST, Cat. #70076), and anti-TNF-α (Proteintech, Cat. #17590-1-AP) were then applied and incubated for 10 h at 4 °C. The slides were treated with polyclonal goat anti-rabbit antibodies labeled with horseradish peroxidase for one hour after being cleaned three times with PBS, followed by chromogen development with diaminobenzidine and staining with hematoxylin. Immunohistochemistry images were captured using an Olympus microscope. The results of IHC staining were assessed using a previously reported semiquantitative method [44,46].

### 3.9. Cell Culture

NIH/3T3 fibroblasts (Cat. #IM-M050) and RAW264.7 macrophages (Cat. #IM-M028) were acquired from Immocell Biotechnology (Xiamen, China). The cells were grown in a 5% CO_2_ and 95% air environment at 37 °C in Dulbecco’s modified Eagle’s medium (DMEM) with 10% FBS until reaching 80% confluence. TGF-β (5 ng/mL) and LPS (100 ng/mL) were included as specified. After 30 min incubation, cells were treated with Co-MnNPs (100 μg/mL), Co-MnNPs (300 μg/mL), or their PBS vehicle for an extra 24 h and collected using trypsin. Collected cells were used for further experiments [37,47].

### 3.10. Real-Time PCR

Total RNA from cells and lung tissue was extracted using the TRIzol reagent. The concentration was measured using a Beckman Coulter spectrophotometer. cDNA was synthesized using wFastKing gDNA dispelling RT SuperMix (TIANGEN, Beijin, China, Cat. #KR170801) and 1mg total RNA following the manufacturer’s method. RT-PCR was performed using SYBR Green PCR Mastermix (Vazyme, Nanjin, China, Cat. #Q111-02) in a 7300 real-time PCR system (Applied Biosystems, Shanghai, China). GAPDH was used as the internal reference gene. Relative quantification (RQ) was calculated based on the difference in cycle threshold (Ct) between the gene of interest and the internal reference genes using the formula: RQ = 2^−ΔΔCT^.

The specific primer sets are presented below:

Fibronectin: 5′-GATGTCCGAACAGCTATTTACCA-3′ (Forward); 5′-CCTTGCGACTTCAGCCACT-3′ (Reverse).

TGF-β: 5′-GCGCTCACTGCTCTTGTGACA-3′ (Forward); 5′-GCAATAGTTGGTATCCAGGGCTCT-3′ (Reverse).

COL1A1: 5′-CCAAGAAGACATCCCTGAAGTCA-3′ (Forward); 5′-TGCACGTCATCGCACACA-3′ (Reverse).

α-SMA: 5′-ACTGGGACGACATGGAAAAG-3′ (Forward); 5′-CATCTCCAGAGTCCAGCACA-3′ (Reverse)

TNF-α: 5′-CACTTGGTGGTTTGCTACGA-3′ (Forward); 5′-GTGGCCCCTGCCACAAGCAG-3′ (Reverse).

IL-1β: 5′-GATCCACACTCTCCAGCTGCA-3′ (Forward); 5′-CAACCAACAAGTGATATTCTCCATG-3′ (Reverse).

IL-6: 5′-CAAAGCCAGAGTCCTTCAGAG-3′ (Forward); 5′-GTCCTTAGCCACTCCTTCTG-3′ (Reverse).

iNOS: 5′-AGGTACTCAGCGTGCTCCAC-3′ (Forward); 5′-GCACCGAAGATATCTTCATG -3′ (Reverse).

GAPDH: 5′-AATGGATTTGGACGCATTGGT-3′ (Forward); 5′-TTTGCACTGGTACGTGTTGAT-3′ (Reverse).

### 3.11. Western Blotting

Extracted lung tissues or NIH/3T3 fibroblasts were lysed with RIPA lysis buffer with inhibitors of phosphatase and protease to acquire protein. The concentration of protein was measured using the BCA kit. 50 ng of proteins was separated using 10% SDS-PAGE gel, then transferred onto BioBond nitrocellulose membranes and incubated at 4 °C for 10 h with the following primary antibodies: anti-p-SMAD2 (CST, Cat. #9681S), anti-p-SMAD3 (CST, Cat. #9520), anti-SMAD2 (Boster, Wuhan, China, Cat. #BA4557), anti-SMAD3 (Boster, Cat. #PB0445), anti-p-TAK1 (Abcam, Shanghai, China, Cat. #ab239974), anti-TAK1 (Boster, Cat. #P01458), anti-p-p38 (Abcam, Cat. #ab195049), anti-p38 (Boster, Cat. #BM4142), anti-p-ERK1/2 (CST, Cat. #9101), anti-ERK1/2 (Proteintech, Cat. #11257-1-AP), anti-p-p65 (Abcam, Cat. #ab32536), anti-a-p65 (Abcam, Cat. #Ab19870), anti-p65 (Abcam, Cat. #ab32536), and anti-GAPDH (Santa Cruz Biotechnology, Dallas, TX, USA, Cat. #sc-47724). After washing off the excess primary antibody, the membrane was treated with the secondary antibody at 25 °C for 2 h. The blot was analyzed using the Image J software 1.45 [48].

### 3.12. Hydroxyproline (HYP) Measurement

The collagen content in pulmonary tissue was examined using an HYP assay kit (Solarbio, Cat. #BC0250). Nearly 100 mg of pulmonary tissue was placed into a test tube and treated with 1 mL of 10 M NaOH. The mixture was digested at 100 °C for 1 h. The samples were then neutralized with HCl to a pH of 6.0–6.8. After 10,000× *g* centrifugation for 5 min, the supernatant was draw and the HYP concentration was measured using an HYP kit. The absorbance was determined using a spectrophotometer at 560 nm, and the amount of HYP was calculated [44].

### 3.13. Statistical Analysis

The results were presented as the mean ± SEM. The statistical analysis and diagram generation were carried out using software GraphPad Prism 9.0.0. For comparing multiple groups, either Student’s *t*-test or one-way analysis of variance (ANOVA) with Tukey’s test was employed. *p* < 0.05 was regarded as statistically significant. 

## 4. Conclusions

In summary, we successfully prepared novel Co-Mn complex oxide nanoparticles (Co-MnNPs) for lung fibrosis treatment. Co-MnNPs can scavenge multiple types of ROS, including •OH and O_2_•^−^. Therefore, Co-MnNPs are able to reduce the generation of ROS in fibroblasts and macrophages, inhibiting the fibrotic and inflammatory responses in these cells through suppressing NF-κB and canonical and non-canonical TGF-β pathways, respectively. Additionally, benefiting from ROS scavenging properties, Co-MnNPs reduce the excessive extracellular matrix deposition and inflammatory responses in lungs and, thus, alleviate pulmonary fibrosis induced by BLM in mice. Taken together, our results indicate that Co-MnNPs are an effective anti-fibrotic agent for pulmonary fibrosis.

## Data Availability

All data are available from the corresponding author upon reasonable request.

## Supplementary Materials

The following supporting information can be downloaded at: https://www.mdpi.com/article/10.3390/molecules29215106/s1, Figure S1: H&E-stained tissue sections of the main organs; Figure S2. TGA assay of Co-MnNPs, Co-Mn and AA; Figure S3. ABTS assay of Trolox; Figure S4. (A) ABTS assay and (B) TMB assay of MSN; Figure S5. MSN has no effects on the activation of (A-B) fibroblasts and (C-D) macrophages; Figure S6. Cytotoxicity of Co-MnNPs (300 μg/mL) in NIH/3T3 and RAW264.7 cells; Figure S7. Immunofluorescent staining for p-TAK1 and α-SMA in HFLs cells; Table S1: PDI assay of Co-MnNPs at pH 5.5 and 7.4.
