# Supplementary material for: Co-Mn Complex Oxide Nanoparticles as Potential Reactive Oxygen Species Scavenging Agents for Pulmonary Fibrosis Treatment
Source: Molecules. 2024 Oct 29;29(21):5106. doi: 10.3390/molecules29215106 (PMC11547682; doi:10.3390/molecules29215106)
Supplement: Supplementary file 1 [file molecules-29-05106-s001.zip › molecules-3204161-supplementary.pdf]

# Co-Mn complex oxide nanoparticles as potential ROS scavenging agents for pulmonary fibrosis treatment

Wuhao Yang [1], Hui Yuan [2, 3], Hao Sun [2, 3], Ting Hu [2, 3], Yaping Xu [4], Yan Qiu [5, 6 *], Yuhang Li [2, 3, 4 *]

1. College of Materials, Xiamen University, Xiamen 361005, China;
2. State Key Laboratory of Structural Chemistry, Fujian Institute of Research on the Structure of Matter, Chinese Academy of Sciences, Fuzhou, Fujian 350002, P. R. China;
3. Xiamen Key Laboratory of Rare Earth Photoelectric Functional Materials, Xiamen Institute of Rare Earth Materials, Haixi Institutes, Chinese Academy of Sciences, Xiamen 361021, P. R. China;
4. Key Laboratory of Functional and Clinical Translational Medicine, Fujian Province University, Xiamen Medical College, Xiamen 361023, China;
5. School of Medicine, Xiamen University, Xiamen, Fujian, 361102, People's Republic of China
6. Xiamen Key Laboratory of Chiral Drugs, Xiamen, Fujian, 361102, People's Republic of China

⁎ Corresponding author: yanqiu@xmu.edu.cn (Y.Q.), yuhangli@fjirsm.ac.cn (Y.L.)

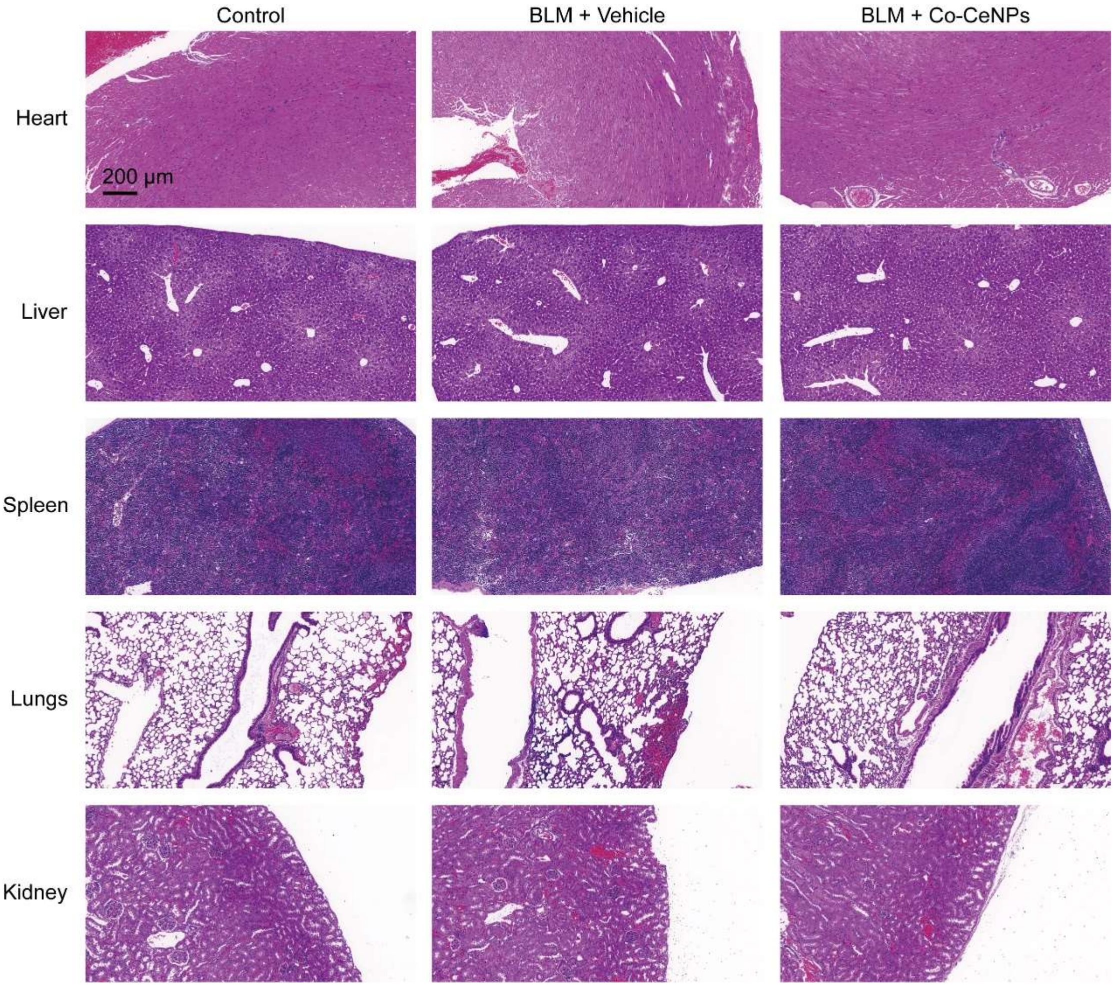


**Figure S1. H&E-stained tissue sections of the main organs**

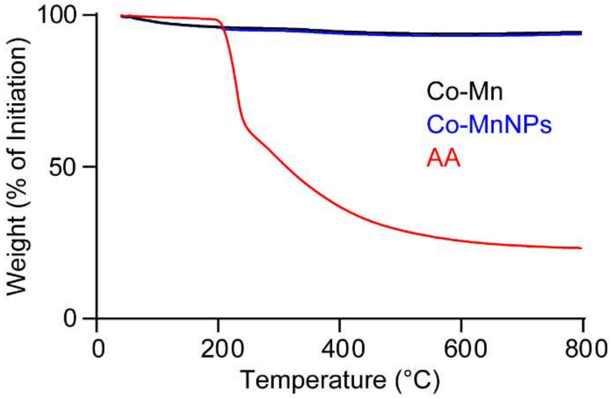


**Figure S2. TGA assay of Co-MnNPs, Co-Mn and AA.**

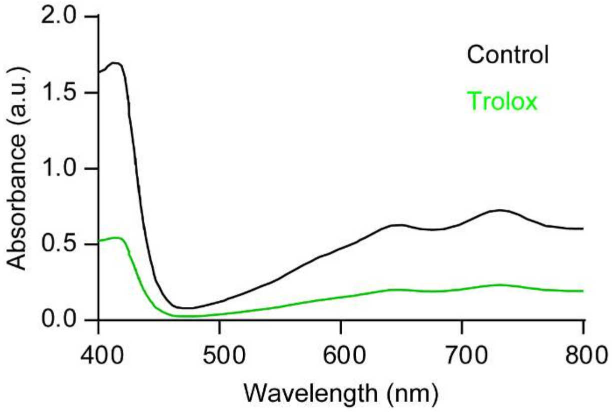


**Figure S3. ABTS assay of Trolox.**

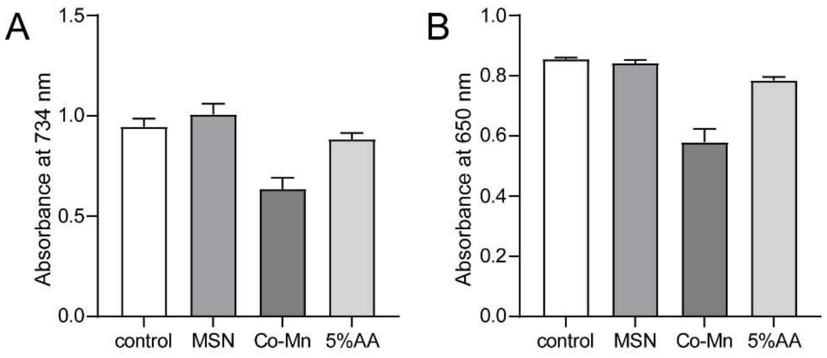


**Figure S4. (A) ABTS assay and (B) TMB assay of MSN.**

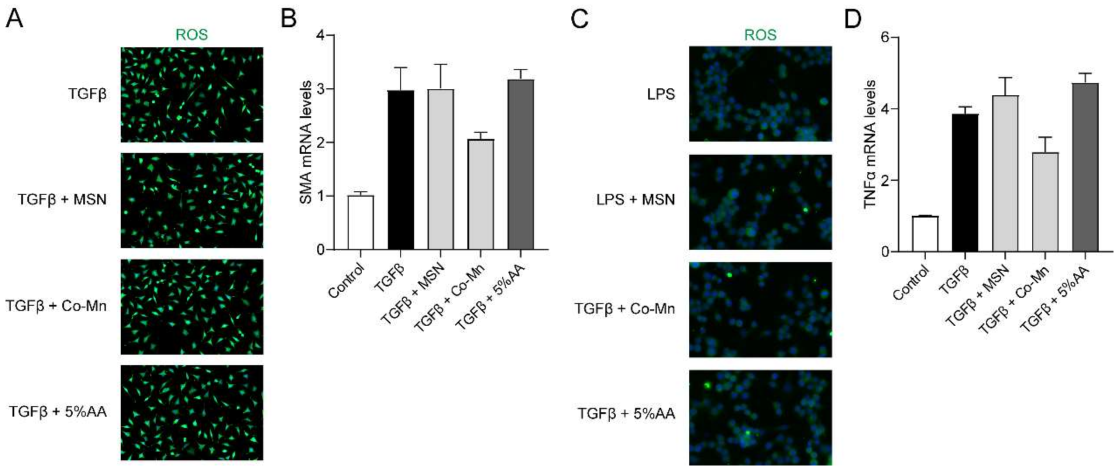


**Figure S5. MSN has no effects on the activation of (A-B) fibroblasts and (C-D) macrophages.**

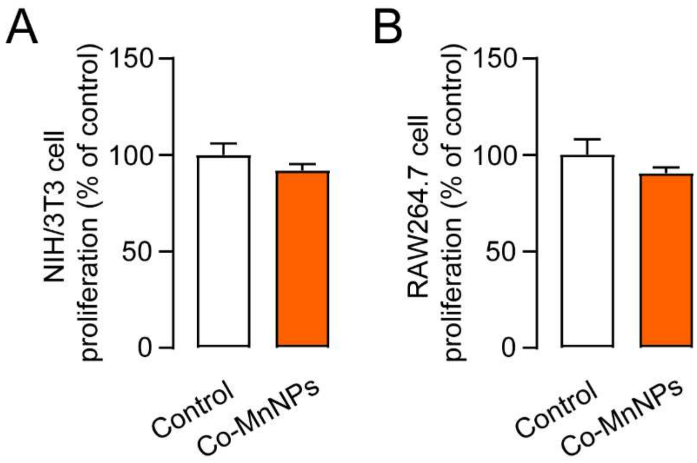


**Figure S6. Cytotoxicity of Co-MnNPs (300 μg/mL) in NIH/3T3 and RAW264.7 cells**

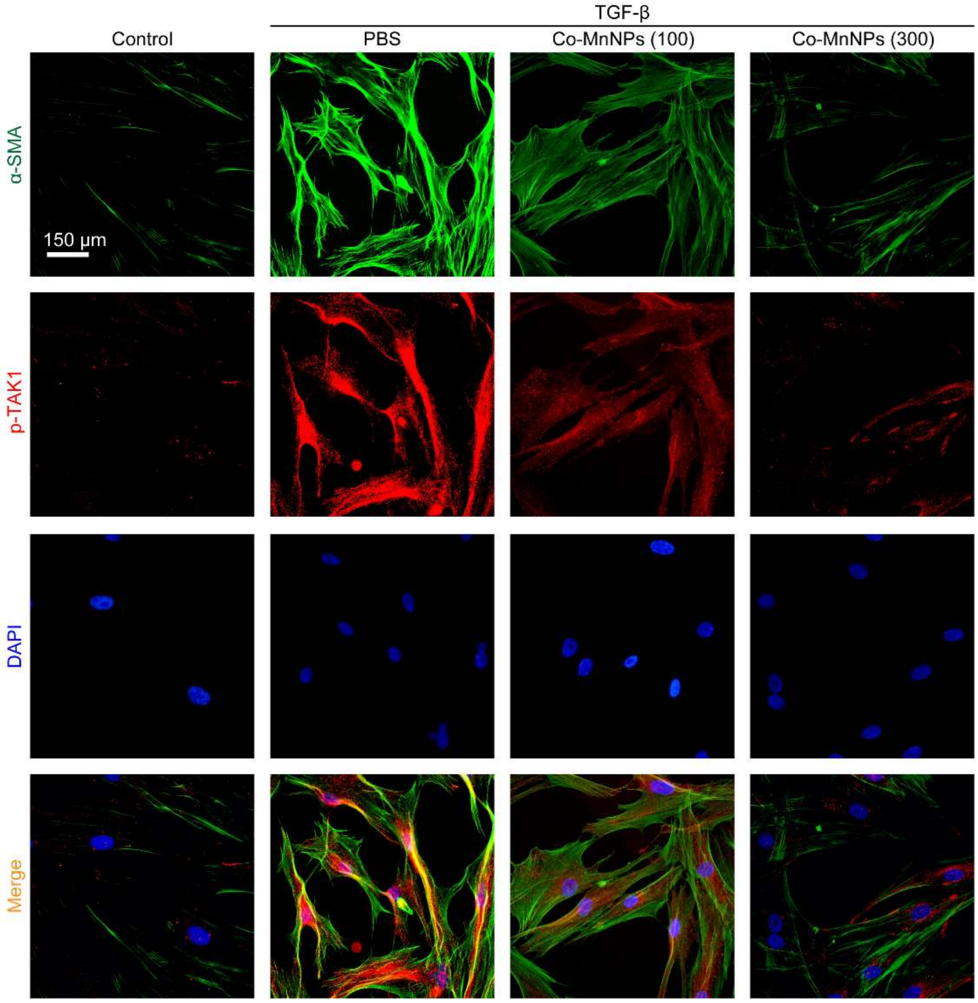


**Figure S7. Immunofluorescent staining for p-TAK1 and α-SMA in HFLs cells**

| | **0 h** | **12 h** | **24 h** | **36 h** | **48 h** |
|---|---|---|---|---|---|
| **pH 5.5** | 0.285 | 0.297 | 0.289 | 0.296 | 0.302 |
| **pH 7.4** | 0.274 | 0.283 | 0.291 | 0.532 | 0.647 |

**Table S1. PDI assay of Co-MnNPs at pH 5.5 and 7.4**
